# The impact of a reduced dose of dexamethasone on glucose control after coronary artery bypass surgery

**DOI:** 10.1186/1475-2840-6-39

**Published:** 2007-12-17

**Authors:** Mathijs Vogelzang, Miriam Hoekstra, José T Drost, Marcel Janse, Iwan CC van der Horst, Piet W Boonstra, Felix Zijlstra, Bert G Loef, Maarten WN Nijsten

**Affiliations:** 1 Department of Cardiology, University Medical Center Groningen, University of Groningen, Groningen, the Netherlands; 2 Surgical Intensive Care Unit, University Medical Center Groningen, University of Groningen, Groningen, the Netherlands; 3 Thoracic Intensive Care Unit, University Medical Center Groningen, University of Groningen, Groningen, the Netherlands; 4 Department of Cardiothoracic Surgery, University Medical Center Groningen, University of Groningen, Groningen, the Netherlands

## Abstract

**Background:**

Intensive insulin therapy to maintain normoglycemia after cardiac surgery reduces morbidity and mortality. We investigated the magnitude and duration of hyperglycemia caused by dexamethasone administered after cardiopulmonary bypass.

**Methods:**

A single-center before-after cohort study was performed. All consecutive patients undergoing coronary artery bypass grafting with cardiopulmonary bypass during a 6-month period were included. Insulin administration was guided by a sliding scale protocol. Halfway the observation period, the dexamethasone protocol was changed. The single dose (1D) group received a pre-operative dose of dexamethasone of 1 mg/kg. The double dose group (2D) received an additional dose of 0.5 mg/kg of dexamethasone post-operatively at ICU admission.

**Results:**

We included 116 patients in the 1D group and 158 patients in the 2D group. There were no significant baseline differences between the groups. Median Euroscore was 5. In univariable analysis, the glucose level was different between groups 1D and 2D at 4, 6, 9, 12 and 24 hours after ICU admission (all p < 0.001). Insulin infusion was higher in the 1D group. Corrected for insulin dose in multivariable linear analysis, the difference in glucose between the 1D and 2D groups was 1.5 mmol/L (95% confidence interval 1.0–2.0, p < 0.001) 12 hours after ICU admission.

**Conclusion:**

Dexamethasone exerts a hyperglycemic effect in cardiac surgery patients. Patients receiving high-dose corticosteroid therapy should be monitored and treated more intensively for hyperglycemic episodes.

## Background

Cardiac surgery with cardiopulmonary bypass induces a strong systemic inflammatory response in previously healthy individuals [1]. This response is characterized by changes in cardiovascular and pulmonary function [2-4]. Corticosteroids inhibit this inflammatory response and may thus ameliorate the adverse effects of cardiopulmonary bypass [1,5,6]. High-dose dexamethasone is one of the therapies given in some centers to reach this effect. However, corticosteroid use in cardiac surgery is controversial, because important side effects may result [7]. One of the side effects is increased insulin resistance, which causes hyperglycemia [8]. The emerging evidence on negative effects of dexamethasone has prompted a protocol change in our institution.

In patients with myocardial infarction, prolonged hyperglycemia after admission is associated with adverse outcome [9,10]. Glycometabolic dysregulation is common in patients undergoing cardiac surgery [11]. Tight glucose control with intensive insulin therapy has been shown to decrease mortality and morbidity in these patients [12]. In patients undergoing coronary artery bypass grafting, the survival benefit persisted at 4 years follow-up [13]. As the benefits of insulin therapy are related to the level of achieved glucose control, the impact of corticosteroids on hyperglycemia has become more relevant [14]. We hypothesized that a reduction of dexamethasone dose would facilitate glucose control. We performed a before-after study to quantify the hyperglycemic effect of a second dose of dexamethasone administered post-operatively at the intensive care unit (ICU) in patients undergoing coronary artery bypass surgery (CABG) with cardiopulmonary bypass.

## Methods

This study was performed at a 14-bed thoracic ICU in a tertiary teaching hospital. During a 6 month period (from April to October 2005) all patients undergoing CABG with the use of cardiopulmonary bypass were studied. Both patients with only CABG and CABG with additional valve repair were included. During the 6-month observation period, the treatment protocol was modified by the medical staff. Before the change, all patients were administered 1.0 mg of dexamethasone per kilogram of body weight at the induction of anesthesia and a second dose of 0.5 mg/kg at admission to the ICU. The patients in this dosing scheme were in the double-dose (2D) group. After the change, the second dose was withheld, so patients only received the initial 1.0 mg/kg dose (group 1D). The protocol change was made after a previous study, in which high-dose dexamethasone failed to provide a beneficial effect on peri-operative renal function [15] and review of other available evidence [7]. The institutional review board approved this study.

At baseline, we collected demographic and clinical information, including sex, age, body mass index (BMI) and presence of diabetes before hospital admission. Operative risk stratification was done by means of the EuroSCORE model [16]. Time on cardiopulmonary bypass and total operation time were recorded. During the ICU stay, we collected blood glucose levels, and insulin administration for the first 48 hours. Glucose levels were measured in arterial blood samples using a point-of-care blood gas analyzer present at the ICU (ABL Radiometer 700 series, Copenhagen). Insulin was administered according to a sliding-scale insulin dosing algorithm. The algorithm was designed to treat hyperglycemia defined as a glucose level over 10 mmol/L but did not aim for normoglycemia (4.4 – 6.1 mmol/L). Our protocol prescribed a 1 liter infusion of glucose 5% during the first 24 hours.

For data processing and statistical analysis SPSS version 12.0 was used. Baseline characteristics of the two groups were expressed as mean ± standard deviation (SD) for normally distributed variables or as median (interquartile range, IQR) for other variables. We compared differences between groups with the Student's t-test for normal distributed variables or the Mann-Whitney U test for other continuous variables. For categorical variables, the Chi-square test or Fisher's exact test were used when appropriate. We analyzed the blood glucose levels at 2, 4, 6, 9, 12, 24 and 48 hours after ICU admission for each patient. The glucose values at these specific times were calculated by linear interpolation. We also calculated the hyperglycemic index for the first 12 hours after admission [17].

The time course of glucose levels was compared with univariable analysis between groups 1D and 2D to determine if and when glucose levels diverged between the two groups. It was then determined at what time the difference in glucose was greatest between the two groups. To assess the relative contributions of patient group (1D or 2D), age, sex, diabetes, BMI and insulin administration to this maximum difference in glucose, we performed multivariable linear regression analysis. We included variables in the model that had a univariable p-value smaller than 0.15. Differences or statistical relations with a p-value of less than 0.05 were considered significant. Bonferroni's correction for multiple testing was applied where appropriate.

## Results

A total of 274 patients were included in this study, 116 in the 1D-group and 158 patients in the 2D-group. Table 1 shows the patient characteristics at baseline for both groups. The mean age of the population was 69 ± 9 years, and 70% of them were male. Median ICU length of stay was 1.0 (0.8–1.8) days and the hospital length of stay was 12 (9–16) days. Pre-operative risk scoring shows that 36% of the population was at medium risk (EuroSCORE 3–5), and 42% was at high risk (EuroSCORE > 5). Thirty-day all cause mortality was 3.3%. The operation time was slightly longer in the 1D group.

**Table 1:** Patient characteristics. Characteristics of the two patient groups. 1D: single dose group, 2D: double dose group. Values are mean ± standard deviation or median (IQR). CPB: cardiopulmonary bypass.

	1D group	2D group	p value
N	116	158	
Male sex	84 (72%)	108 (68%)	NS
Age (years)	68 ± 10	69 ± 8	NS
Diabetes mellitus	33 (28%)	36 (23%)	NS
Body mass index	27.4 ± 3.4	27.6 ± 3.9	NS
EuroSCORE	5 (2 – 7)	5 (3 – 7)	NS
Other than isolated CABG	32 (28%)	33 (21%)	NS
Emergency procedure	3 (3%)	7 (5%)	NS
Number of anastomoses	3 (2 – 4)	3 (2 – 4)	NS
Time on CPB (hours)	1.8 (1.3 – 2.7)	1.6 (1.4 – 2.3)	NS
Operation time (hours)	5.5 (4.6 – 6.4)	5.0 (4.3 – 5.9)	.01
30-day mortality	3 (2.6%)	6 (3.8%)	NS

**Figure 1:**
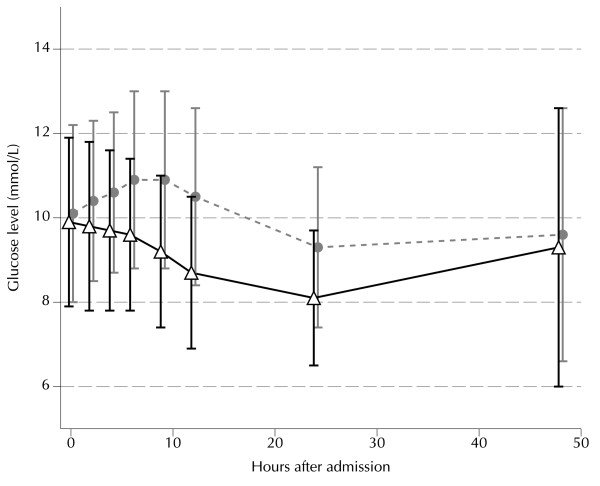
**Time course of glucose levels after ICU admission**. Comparison of post-cardiac surgery hyperglycemia for the single dose group (1D group, triangles, 116 patients) and for the double dose group (2D group, circles, 158 patients). Data are medians and interquartile ranges. The glucose level was higher at 4, 6, 9, 12, and 24 hours in the 2D group (all p < 0.001, after Bonferroni correction for multiple testing). The difference was largest at 12 hours after ICU admission.

**Figure 2:**
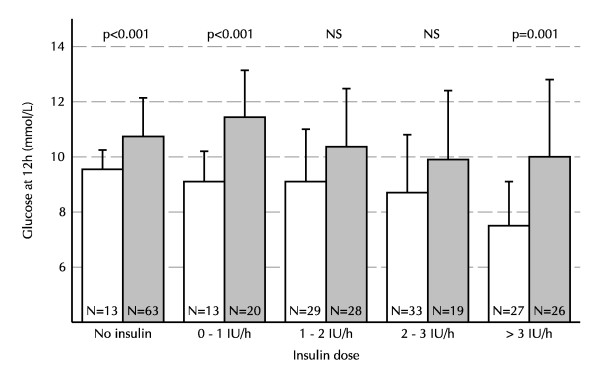
Glucose levels at 12 hours post ICU admission stratified for mean insulin dose before that time. White bars represent the 1D group, gray bars represent the 2D group.

Figure 1 shows the mean and standard deviation of blood glucose levels over the first 48 hours after ICU admission. At 4, 6, 9, 12 and 24 hours after admission glucose levels were significantly lower in the 1D-group than in the 2D-group, with the greatest difference at 12 hours. The hyperglycemic index over the first 12 hours was significantly lower in the 1D group: 3.5 ± 1.4 mmol/L vs. 4.7 ± 1.7 mmol/L (p < 0.001). Performance and attention for the glucose control protocol improved during the study period. Patients in the 1D group received 26 (15 – 36) units of insulin during the first 12 hours of ICU stay, for the 2D group this was 12 (0 – 27.25, p < 0.001). The number of glucose measurements in the first 12 hours was 6 (4 – 7) in the 1D group and 4 (3 – 5) in the 2D group. Figure 2 shows the glucose level 12 hours after admission for both groups stratified for mean insulin infusion rate during the first 12 hours. In multivariable linear regression analysis, the attributable increase of glucose at 12 h after admission was 1.5 mmol/L for a second dose of dexamethasone (p < 0.001, table 2). The difference in glucose levels between the 1D and 2D groups was similar in patients with and without a history of diabetes.

**Table 2:** Linear analysis of determinants of glucose level. Parameters in the linear model predicting glucose level 12 hours after admission. CI: confidence interval. *: p < 0.05. +: p < 0.001.

Parameter	Univariable coefficient (95% CI)	Multivariable coefficient (95% CI)
Age (per year)	0.04 (0.01 to 0.07)^+^	0.05 (0.03 to 0.07)^+^
Male sex	NS	
History of diabetes	NS	
Body mass index	NS	
Other than isolated CABG	-0.63 (-1.25 to -0.01)*	NS
Emergency procedure	NS	
EuroSCORE	NS	
Time on CPB	-0.51 (-0.83 to -0.19)^+^	NS
Operation time	-0.32 (-0.51 to -0.13)^+^	NS
Insulin in the first 12 hours (units)	-0.033 (-0.045 to -0.021)^+^	-0.024 (-0.036 to -0.012)^+^
2D vs 1D group	1.8 (1.3 to 2.3)^+^	1.5 (1.0 to 2.0)^+^

## Discussion

In this study we found that cardiac surgical patients who received two doses of dexamethasone displayed higher blood glucose levels between 6 and 24 hours after ICU admission than patients who received one dose of dexamethasone.

High dose dexamethasone is a controversial therapy in cardiac surgery patients. Both studies that favor the use of corticosteroids [5] or studies that show some benefit [6] as well as studies that show no benefit [7,15,18] have been published. The effect of steroids on glucose control may be of interest, because high glucose levels are common and have been shown to be independent predictors of adverse outcome during cardiac surgery [11,19]. Treatment of hyperglycemia by continuous insulin infusion has recently been shown to improve outcome [12,13,20]. The difference of 1.5 mmol/L in glucose levels we found in multivariable analysis is clinically relevant considering a post-hoc analysis found that the odds ratio for ICU mortality for every increase in glucose level of 1.1 mmol/L is 1.3 [14]. Our study was not powered to detect a difference in mortality.

While the merit of steroids for cardiopulmonary bypass may be disputed, steroids are administered for many other indications in other critically ill patients. For instance, lower dose corticosteroids may be used to prevent atrial fibrillation after cardiac surgery [21]. For other patient categories, effects on glucose control have recently been shown for hydrocortisone in patients with septic shock [22], or for dexamethasone against nausea in abdominal surgery patients [23].

A number of limitations of our study must be mentioned. First, we had not implemented a tight glucose control protocol at the thoracic ICU at the time of this study. Therefore, overall glucose control was not comparable to the levels achieved in intervention studies, reflected by the HGI which indicates mean glucose levels of 9.5 and 10.7 mmol/L for the groups respectively. We think this does not change our conclusion that dexamethasone induces hyperglycemia, as we hypothesize that under a tight glucose control protocol, we would have found that the 1D group would require less interventions and lower doses of insulin than the 2D group to achieve the same glucose targets. Second, although data collection was prospective, the study as a whole must be regarded as a retrospective study. Bias could have been introduced in our data in a number of ways. Inherent to the before-after design the patients were not randomly allocated to the two dosing groups. A very important variable, insulin infusion, was found to be significantly different between the two groups. The multivariable analysis incorporating this and other possible confounding factors confirmed the highly significant relation of glucose levels with the administration of one or two dexamethasone doses. This analysis may still overestimate the difference caused by dexamethasone due to unknown confounding factors and due to non-linear effects of the included variables. Not all cardiac surgery centers use dexamethasone as routine treatment in all cardiac surgery patients. This may be a limitation to the relevance of our study. However, it is unlikely that the hyperglycemic effect we measured in this study is unique to dexamethasone or cardiac surgery patients. Our findings may be extrapolated to the majority of patients receiving corticosteroids in the intensive care setting.

Although we did not study the effect of giving dexamethasone compared to no dexamethasone is this study, the first dose of dexamethasone also induced considerable insulin resistance as we have observed in a previous study [18]. Withholding of the dose administrated upon induction of anesthesia could further improve glucose control around the time of ICU admission. This quantitative study shows that one should be prepared to administer higher doses of insulin when using dexamethasone in cardiac surgery patients. In three ICUs in our hospital, we have implemented a computerized glucose control system. We designed this system so that patients who receive steroids both get higher initial insulin doses, and are checked more frequently to promptly detect hyperglycemia [24]. Future research on the value of corticosteroids during cardiac surgery or for other indications in the ICU should pay very close attention to the hyperglycemic effects of these drugs. If glucose control is not performed adequately, potential positive effects of steroids could be offset by iatrogenic hyperglycemia.

In summary, the administration of dexamethasone exerts a considerable hyperglycemic effect in cardiac surgery patients, interfering with glucose control.

## Competing interests

The author(s) declare that they have no competing interests.

## Authors' contributions

All authors were involved in drafting the manuscript. All authors read and approved the final version. MH, JD, MJ and MN collected data. PB provided EuroSCORE data. MV, IvdH, FZ, BL and MN designed the study. MV and MN performed statistical analyses. MN coordinated the study.
